# Community-directed distributors—The “foot soldiers” in the fight to control and eliminate neglected tropical diseases

**DOI:** 10.1371/journal.pntd.0009088

**Published:** 2021-03-04

**Authors:** Uche V. Amazigo, Stephen G. A. Leak, Honorat G. M. Zoure, Chukwu Okoronkwo, Maimouna Diop Ly, Sunday Isiyaku, Andy Crump, Joseph C. Okeibunor, Boakye Boatin

**Affiliations:** 1 African Programme for Onchocerciasis Control, World Health Organization, Enugu, Nigeria; 2 African Programme for Onchocerciasis Control, World Health Organization, Macclesfield, Cheshire, United Kingdom; 3 World Health Organization, Regional Office for Africa, Brazzaville, Congo; 4 Federal Ministry of Health, Abuja, Nigeria; 5 African Development Bank, Abidjan, Côte d’Ivoire; 6 Sightsavers, Nigeria Country Office, Kaduna, Nigeria; 7 Kitasato University, Tokyo, Japan; 8 World Health Organization, Regional Office for Africa, Brazzaville, Congo; 9 Onchocerciasis Control Programme in West Africa, World Health Organization, Accra, Ghana; Task Force for Global Health, UNITED STATES

## Abstract

The neglected tropical diseases (NTDs) affect hundreds of millions of people, predominantly in rural, often difficult-to-access areas, poorly served by national health services. Here, we review the contributions of 4.8 million community-directed distributors (CDDs) of medicines over 2 decades in 146,000 communities in 27 sub-Saharan African countries to control or eliminate onchocerciasis and lymphatic filariasis (LF). We examine their role in the control of other NTDs, malaria, HIV/AIDS interventions, immunisation campaigns, and support to overstretched health service personnel. We are of the opinion that CDDs as community selected, trained, and experienced “foot soldiers,” some of whom were involved in the Ebola outbreak responses at the community level in Liberia, if retrained, can assist community leaders and support health workers (HWs) in the ongoing Coronavirus Disease 2019 (COVID-19) crisis. The review highlights the improved treatment coverage where there are women CDDs, the benefits and lessons from the work of CDDs, their long-term engagement, and the challenges they face in healthcare delivery. It underscores the value of utilising the CDD model for strong community engagement and recommends the model, with some review, to hasten the achievement of the NTD 2030 goal and assist the health system cope with evolving epidemics and other challenges. We propose that, based on the unprecedented progress made in the control of NTDs directly linked to community engagement and contributions of CDDs “foot soldiers,” they deserve regional and global recognition. We also suggest that the World Health Organization (WHO) and other international stakeholders promote policy and guidance for countries to adapt this model for the elimination of NTDs and to strengthen national health services. This will enhance the accomplishment of some Sustainable Development Goals (SDGs) by 2030 in sub-Saharan Africa.

## Author summary

Community-directed distributors (CDDs), sometimes known as community health workers (CHWs), have proved to be critical in the delivery of medicines and other tools for the control of neglected tropical diseases (NTDs), prevention of malaria, and other beneficial health interventions. The distributors are the unsung heroes and heroines without whom the health of hundreds of thousands of communities in rural Africa would be worse than it is today. In this paper, we document more than 2 decades (1997–2019) of the contributions of 146,000 communities and 4.8 million CDDs of medicines for NTDs, unpaid or minimally compensated, some have provided 18 years of uninterrupted service. We report on the burden of work and their perspectives of the challenges involved in mass drug administration (MDA) across 27 countries in sub-Saharan Africa. We suggest that they have not been adequately recognised and that harnessing such community human resources could contribute to improving health system’s responses to the ongoing Coronavirus Disease 2019 (COVID-19) crisis. We recommend policy measures for a wider application of existing networks of CDDs by countries’ health systems to consolidate and accelerate the achievements made as well as for the attainment of the goals set forth in the newly developed World Health Organization (WHO) NTD Roadmap.

## Introduction

The Member States of the World Health Organization (WHO) have recognised health equity as a basic human right and the core value of universal health coverage (UHC). The goal of UHC is to ensure that everyone attains his or her full health potential and has equitable, barrier-free access to healthcare, regardless of social position or circumstances. The “how” to accomplish this health equity goal is the question now confronting the international community.

The framework on integrated people-centred health services (IPCHS) aims to address these issues by calling for a fundamental shift in the way health services are funded, managed, and delivered [1]. IPCHS will necessitate putting the comprehensive and holistic needs of people and communities, not only diseases, at the centre of health systems, and empowering individuals to have a more active role in determining and satisfying their health needs.

Policy makers and key stakeholders in health remain inadequately informed of the contributions of community-directed distributors (CDDs) towards the delivery of medicines and other tools in the fight against onchocerciasis (river blindness), lymphatic filariasis (LF), and other neglected tropical diseases (NTDs), along with malaria, tuberculosis (TB), and HIV/AIDS, in Africa. The CDDs are the unsung heroes and heroines without whom the health of hundreds of thousands of communities in rural Africa would be worse than it is today. Indeed, some of the achievements of the Millennium Development Goals (MDGs) undoubtedly profited from the selfless commitment and substantive contribution of communities and the unpaid or minimally compensated CDDs [2].

The fight against some of the world’s most devastating, disfiguring, and socioeconomically damaging and stigmatising NTDs has engendered an unprecedented global response. Diverse public and private sector stakeholders are contributing to what is recognised as being one of the world’s most remarkable public health success stories [3] in which the CDDs play a key role. Global elimination of LF and onchocerciasis is now possible within a reasonable time frame [4,5]. In 2017 and 2019, Togo and Malawi achieved the elimination of LF as a public health problem, respectively [6,7]. Uganda and 3 states in Nigeria (Plateau, Nassarawa, and Kaduna) have interrupted the transmission of onchocerciasis [8–10]. In addition, Ghana has achieved the elimination of trachoma as a public health problem [11]. These countries/programmes have relied on or were built predominantly on the work of CDDs for these achievements.

The concept of using community members as community health workers (CHWs) began in China with Farmer Scholars in the 1930s and the barefoot doctors initiative in the 1950s [12,13]. In Africa, with the donation of ivermectin (IVM; Mectizan) by Merck in 1987, various national and international non-governmental development organizations (NGDOs) and national Ministries of Health pioneered community-based strategies for IVM distribution in Mali and Nigeria in the 1990s [14]. The achievements were further advanced with the development of the community-directed treatment (CDT) with (IVM) methodology and the successes of programmes targeting single diseases, such as LF [15,16] and onchocerciasis. The school-based initiatives (SBIs) against schistosomiasis and soil-transmitted helminths (STHs) have also recorded successes with local teachers delivering donated drugs [17]. Recent approaches promoted the integration of CDT, SBI, and other health system programmes in an intensified effort to overcome the diverse and reemerging health challenges in Africa.

For primary health care (PHC) disease control programmes, it is currently accepted that they should be rooted in communities and allow the affected communities to make or influence decisions which impact their own healthcare [18,19]. This key principle of the 1978 Alma Ata Declaration, plus the initial NGDO-driven community-based treatment in Mali, guided the research and was effectively used to harness available resources and the workforce at the community level in Africa [20].

CHWs for other health interventions were in operation in some African countries before the African Programme for Onchocerciasis Control (APOC) was established in 1995. However, no such initiative using CDDs as a massive workforce for the delivery of donated medicines on a large scale existed in sub-Saharan Africa until then. CDDs volunteer their time, starting in 1997 as “foot soldiers,” complementing the work of health personnel and ensuring that remote communities were finally connected to national health services. The communities select their CDDs and determine means of supporting them either in cash or in kind. This approach confirmed that the CDT strategy [20] of devolving some healthcare responsibilities to communities and the CDDs they chose would compensate for the dearth of health workers (HWs) at the community level that was and still is commonplace in sub-Saharan Africa, especially in hard-to-reach areas.

CDDs have received little recognition over time for their contributions and comparatively no remuneration for their efforts. They are, essentially, the poor relations in a diverse family, despite their vital and indispensable role in the control/elimination of NTDs. In this paper, we document more than 2 decades (1997 to 2019) of the contributions of over 146,000 communities and 4.8 million CDDs and their perspectives of the challenges involved in mass drug administration (MDA) across 27 countries in sub-Saharan Africa. We analysed the contribution of women in the MDA process and identified lessons learned from engaging communities and CDDs in large-scale distribution of medicines and tools in the fight against NTDs. The data sources of this paper are quantitative and qualitative data submitted to APOC annually by the national onchocerciasis taskforces (NOTFs) of endemic countries and NGDOs that were part of the Onchocerciasis Control Programme (OCP) in West Africa and APOC, NTD managers, and a review of 74 papers from the literature.

The paper recommends policy measures and provides technical guidance to NTD programmes on the roles and responsibilities of communities, CDDs, and countries in integrated NTD programming to improve performance. Furthermore, it envisages a wider application of existing networks of CDDs by countries’ health systems to accelerate the achievements and revised goals of the WHO NTD Roadmap of 2030.

## Why engagement of community-directed distributors became necessary

Despite the availability of many effective disease control medicines, these have had limited impact on the burden of disease because of poor access and weak delivery systems.

Following the confirmation of IVM as a safe and effective drug for treating onchocerciasis and Merck’s decision to donate the drug in 1987 [3], the greatest challenge was to make the drug accessible to populations in hard-to-reach and often security-compromised communities where it was most needed. It was evident that the prevention and treatment of diseases, in particular NTDs, could not be left to health systems which, in Africa, had fewer than 23 doctors and nurses for a population of 10,000 even in the best scenario [21]. Thus, this necessitated the harnessing of community human resources which could become an extension of the health service in the delivery of drugs such as IVM to those that really needed them.

The at-risk population in the onchocerciasis endemic communities in 27 countries (except for Gabon, for which data are for 2004 and Angola and the Central African Republic [CAR], for which data are for 2012) was estimated to be 118,100,000 and 169,196,267 in 1995 [22] and 2013 [23], respectively, predominantly located in hard-to-reach terrains. To treat the target population annually, a cumulative total of 555,743 HWs were trained over the 14-year period (2000 to 2013) for annual treatments of communities in need.

For the purposes of this paper, HWs are considered to be trained persons employed by national health systems rather than volunteer community members who may not have formal qualifications in healthcare. The ratio of trained HWs available to treat the at-risk communities during MDA with IVM, both door to door and from central points, in the 27 countries each year was very low. For example, in 2013, the ratio of trained HWs to the treated population in Cameroon was 1 HW:2,164 people treated; in Chad, the ratio was 1:10,244. These figures were well above the recommended ratio of 23 HW:10,000 population (or 1 HW:435 persons) [21,24]. These data demonstrate the importance of the essential role of CDDs in enabling the distribution of IVM to take place successfully and at levels higher than could be accomplished by HW alone.

The enormity of the task of the health system is better appreciated when the ratio of trained HWs is juxtaposed on the geographical spread of the communities in mostly inaccessible locations. Tables 1 and 2 indicate the low ratio in 5 countries of HWs (ranging from medical doctors to nurses), in relation to the populations at risk of NTDs generally and onchocerciasis specifically. Using the UHC index, an indicator of access to essential health services used to assess one of the Sustainable Development Goals (SDGs), the indices for developed countries, such as the United Kingdom, France, the Netherlands, Canada, and the United States of America, are all >80 compared to those NTD endemic countries in Africa (Table 2). The UHC index is, according to the WHO definition, an index, based on a unitless scale of 0 to 100 computed as the geometric mean of 14 tracer indicators of health service coverage [24]. The data in Tables 1 and 2 thus illustrate the significant contribution CDDs can, and are able to, make towards improving healthcare, especially for remote rural communities and the indispensability of the communities themselves selecting the community-based “foot-soldiers,” the CDDs.

**Table 1:** Numbers of HWs and CDDs trained in representative countries of the APOC programme in 2013.

Country	Population at risk of onchocerciasis	Number of HWs trained	Number of CDDs trained
Angola	1,212,200	346	3,819
Cameroon	7,742,400	3,055	45,390
DR Congo	33,377,800	7,942	117,575
Ethiopia	9,654,560	9,090	98,546
Ghana	4,279,086	5,162	7,532
Nigeria	42,265,675	39,250	166,842
Tanzania	2,444,067	1,635	13,395
Togo	3,094,350	742	8,829

APOC, African Programme for Onchocerciasis Control; CDD, community-directed distributor; HW, health worker.

**Table 2:** Health statistics for selected APOC countries related to the NTDs.

Country	Total population (2016)	Population requiring intervention against NTDs (2016 figures)	Life expectancy (2016)	Healthy life expectancy at birth (2016)	UHC index (2015)
Angola	28,813,000	14,419,092	62.6	55.8	36
Cameroon	23,439,000	19,389,766	58.1	51.1	44
DRC	25,369,000	49,900,757	60.5	52.5	40
Ethiopia	102,403,000	74,204,513	65.5	57.5	39
Ghana	28,207,000	15,536,910	63.4	56.4	45
Nigeria	185,990,000	128,936,746	55.2	48.9	39
Tanzania	55,572,000	25,008,679	63.9	56.5	39
Togo	7,606,000	6,328,077	60.6	53.9	42

Source: WHO statistics for 2018.

APOC, African Programme for Onchocerciasis Control; DRC, Democratic Republic of Congo; NTD, neglected tropical disease; UHC, universal health coverage; WHO, World Health Organization.

This information is based on 2013 APOC data except for Angola, for which the figures are for 2012.

See S1 Data on the data used for Table 1.

## Community-directed distributors and ivermectin distribution

Between 2000 and 2013, 27 African countries had almost 5 million (4,928,920) trained CDDs. However, there is inevitably attrition, as some CDDs who have been trained cease to carry on working. This occurs for a variety of reasons, such as moving to live in other areas, or simply deciding that they no longer want to continue with their voluntary work. The number of CDDs trained by the health facility staff and provided with NTD medicines to distribute is recorded and reported to the district and national programme office annually. CDDs who fail to participate in the training or drug collection in preparation of a treatment cycle are counted as dropouts. CDDs who wish to stop their CDD work often inform the community leader or HW before the next treatment to enable replacements to be found and trained. The NTD project records information about attrition of CDDs, if any, annually. The community leadership has the responsibility of finding replacements.

As shown in Table 3, the number of CDDs available over the 14-year period was 4,781,181. The rates of attrition varied among countries, and in few, such as CAR and Ghana, the attrition rate of trained CDDs was relatively high (Table 3). But for the data gap on availability of CDDs in few countries for the period 2000 to 2008, the cumulative number of available CDDs should be greater than or equal to the cumulative number of CDDs trained since not all previously trained CDDs are systematically retrained every year.

**Table 3:** Distribution of CDDs and HWs trained and available between 2000 and 2013*.

Subregion	Country	Period of APOC data submission*	Cumulative no. of CDDs trained	Cumulative no. of CDDs available**	Cumulative no. of HWs trained
**West Africa**	Benin	2011–2013	29,410	29,410	837
	Burkina Faso	2011–2013	5,151	5,293	381
	Côte d’Ivoire	2008–2013	31,087	34,188	1,949
	Ghana	2008–2013	32,880	14,443	9,697
	Guinea	2011–2013	25,641	22,079	521
	Guinea-Bissau	2008–2013	3,219	2,524	82
	Liberia	2000–2013	104,638	99,913	5,601
	Mali	2011–2013	24,978	24,946	1,466
	Nigeria	2000–2013	1,521,595	1,350,791	268,222
	Senegal	-	Not available	Not available	Not available
	Sierra Leone^#^	2009–2013	93,972	80,448	4,737
	Togo	2011–2013	15,773	15,773	1,395
***Total West Africa***			***1*,*888*,*344***	***1*,*679*,*808***	***294*,*888***
**Central Africa**	Angola	2005–2012	18,630	18,971	2,098
	Burundi	2005–2013	67,734	68,042	1,732
	Cameroon	2000–2013	338,130	359,269	31,593
	CAR	2000–2012	62,867	20,450	5,221
	Chad	2000–2013	42,515	95,197	2,773
	Congo	2001–2013	23,383	23,344	2,271
	DRC	2001–2013	829,581	882,704	60,197
	Eq. Guinea	2000–2013	1,748	1,748	150
	Gabon	2002–2003	0	0	20
***Total Central Africa***			***1*,*384*,*588***	***1*,*469*,*725***	***106*,*055***
**East Africa**	Ethiopia	2001–2013	628,787	648,469	54,848
	Malawi	2000–2013	118,129	121,780	23,966
	South Sudan	2000–2013	78,986	79,992	5,614
	Sudan	2000–2013	23,508	22,813	9,847
	Tanzania	2000–2013	129,658	130,066	10,591
	Uganda	2000–2013	676,920	628,528	49,934
***Total East Africa***			***1*,*655*,*988***	***1*,*631*,*648***	***154*,*800***
**Grand total**			**4,928,920**	**4,781,181**	**555,743**

Source: WHO/APOC [22,23] and S1 Data on the data used for Table 3.

* The data collection period varies for country according to the year a country started to submit data to APOC.

** Cumulative no. of CDDs available may also include those present before the training from 2000 to 2013 in the countries.

# Sierra Leone: Data for CDDs were only available from 2010 to 2013. Data for HWs were from 2009 to 2013.

APOC, African Programme for Onchocerciasis Control; CAR, Central African Republic; CDD, community-directed distributor; DRC, Democratic Republic of Congo; HW, health worker; WHO, World Health Organization.

Nonetheless, a significant proportion of CDDs in the 27 countries continued to serve their communities for long periods as illustrated for 3 countries in Table 4. For example, data from Nigeria (shown in Table 4) confirm that in 23 local government areas of Kaduna State, 1,521 CDDs served for the entire 10-year period from 2009 to 2018. Of this total, 1,369 were male, and 152 were female. About half of those CDDs had served since the year 2000 (18 years). In Ghana, 9,131 CDDs including a high proportion of females served their communities for 10 years (2009 to 2018). And almost one-third of those (2,827) served for 18 years (2000 to 2018) in the 9 regions with MDA (Table 4).

**Table 4:** CDDs who have served in MDA uninterrupted for 10 and 19 years in Ghana, Kaduna State, Nigeria, and Cameroon (southwest region) between 2000 and 2018.

Country	CDDs who have served from 2009 to 2018					CDDs who have served from 2000 to 2018				
	Male	%	Female	%	Total	Male	%	Female	%	Total
**Ghana**	6,951	76.1%	2,180	23.9%	9,131	2,497	88.3%	330	11.7%	2,827
**Nigeria (Kaduna State)**	1,369	90.0%	152	10%	1,521	637	87.6%	90	12.4%	727
**Cameroon (southwest region)**	No data					406	51.8%	378	48.2	784
**Overall total**	**8,320**	**78.1%**	**2,332**	**21.9%**	**10,652**	**3,540**	**81.6%**	**798**	**18.4%**	**4,338**

CDD, community-directed distributor; MDA, mass drug administration.

In the 18 districts of the southwest region of Cameroon, data are unavailable for 2009 to 2018. However, 784 CDDs of which almost half (48.2%) are females have served for 18 years (Table 4). See S1 Table for further information on the data used for Table 4.

## Network and number of CDDs afield

Between 2000 and 2013, national Ministries of Health and their partners (APOC, the Global Alliance for the Elimination of Lymphatic Filariasis [GAELF], and NGDOs) trained almost 5 million (4,928,920) community members as CDDs of which 4,781,181 actively participated in preventive chemotherapy activities in 27 countries during the same period (Table 3).

Women constituted 919,292 (19%) of the CDDs in 27 countries. Nigeria had the largest number of CDDs 1,521,595 trained, while 1,350,791 were available for the work. Some 27% (364,013) of the available CDDs in Nigeria were females. The Democratic Republic of Congo (DRC) had cumulative totals of 829,581 CDDs trained and 882,704 CDDs available (Table 3). The sex of the CDDs was only recorded consistently by community-directed treatment with ivermectin (CDTI) projects from 2009 onwards, and of the cumulative total of 580,007 CDDs for whom sex was recorded, 126,141 (22%) were women.

**Fig 1:**
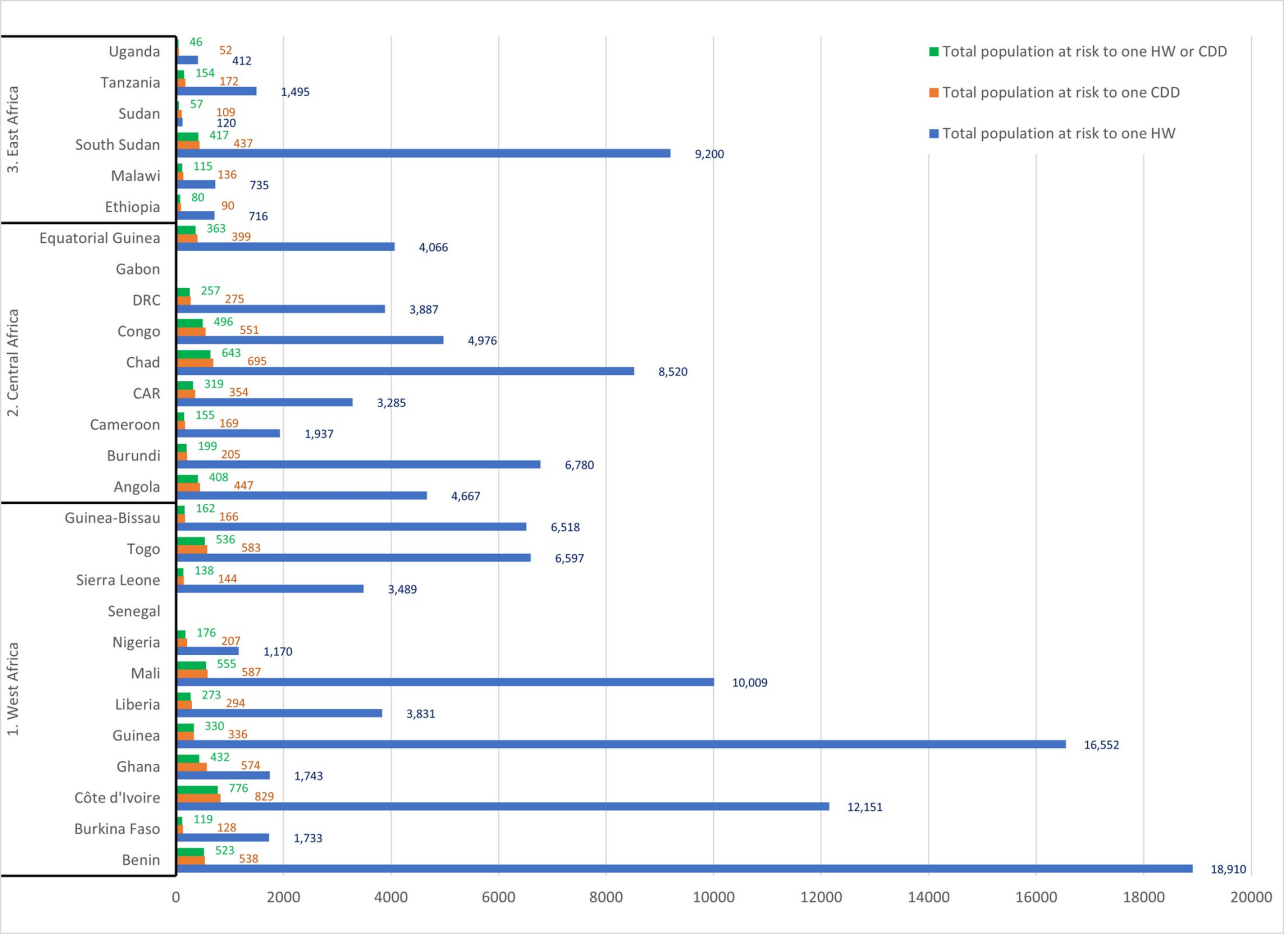
Reduction of population at risk in relation to number of trained HWs and CDDs by country (1997–2013). CAR, Central African Republic; CDD, community-directed distributor; DRC, Democratic Republic of Congo; HW, health worker.

**Fig 2:**
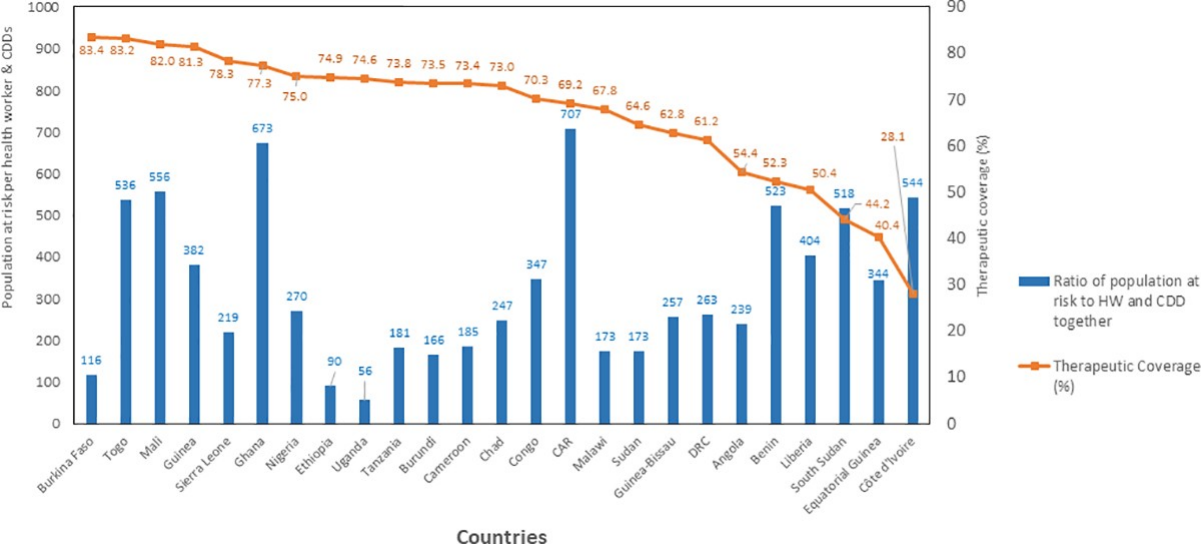
Therapeutic coverage and population at risk per trained HWs and CDDs by country. CAR, Central African Republic; CDD, community-directed distributor; DRC, Democratic Republic of Congo; HW, health worker.

**Fig 3:**
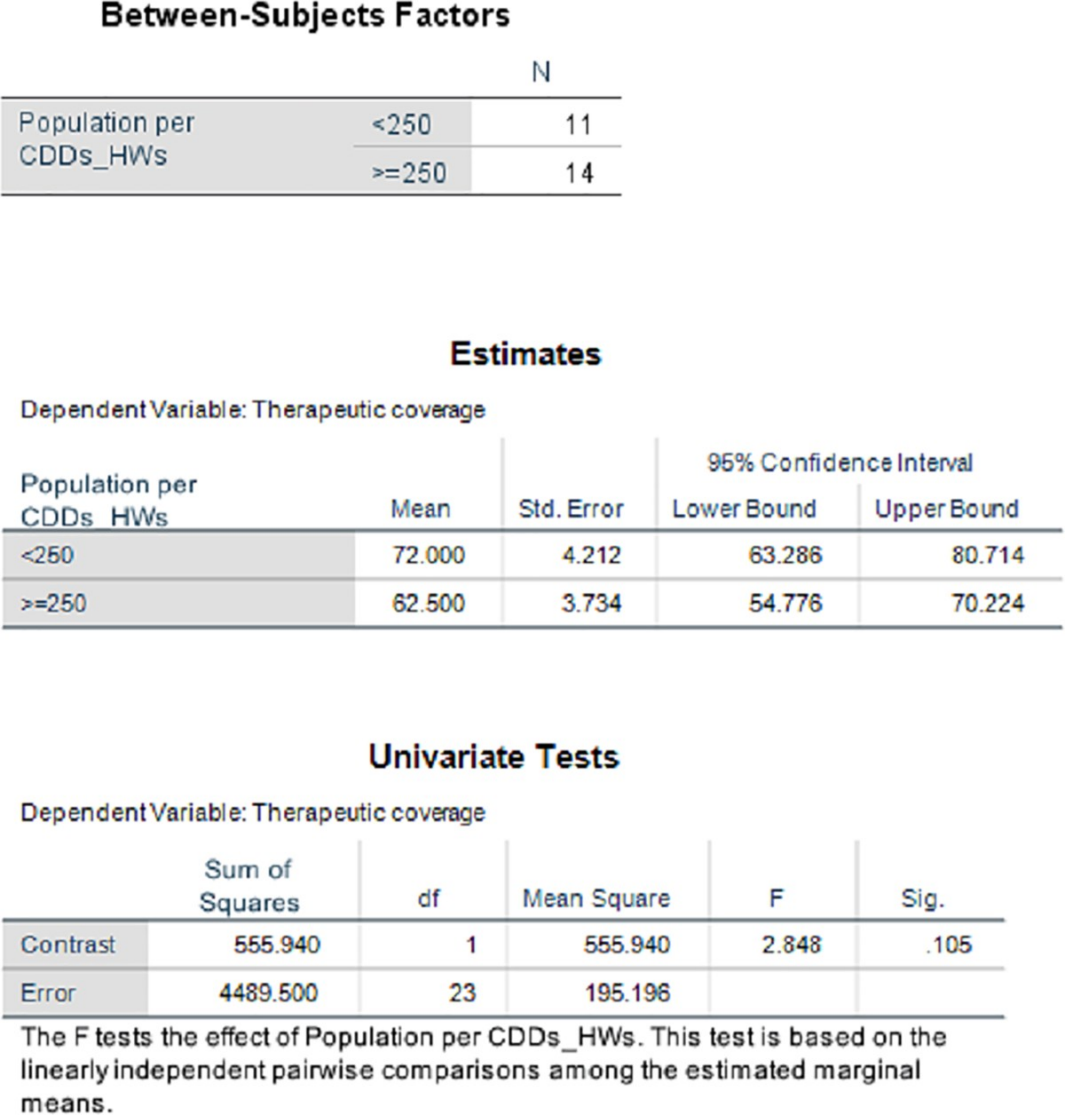
Results of the comparison of mean therapeutic coverage between population per CDD/HW ratio. CDD, community-directed distributor; HW, health worker.

Availability of CDDs significantly improved the ratio of distributors of IVM for the prevention of river blindness and LF from the 2,123 persons at risk per 1 HW to a low average of 247:1 trained IVM distributor. In countries with high ratios in excess of 10,000, such as Côte d’Ivoire and Mali (Fig 1), the training of CDDs brought the ratio down significantly to 829 and 587, respectively. When combined with the trained HWs, the ratios in these countries were further reduced to 544:1 for Côte d’Ivoire and 556:1 for Mali (Figs 2 and 3).

See S1 Data on the data used for Tables 1 and 3 and Figs 1–3.

After the landmark London Declaration in 2012 [25], an increased number of pharmaceutical companies continued to take full responsibility for the costs and logistics for delivering the required number of donated medicines to a port of entry in a recipient nation. With financial support from donors, individual governments and NGDOs take responsibility, including financing, for delivering the medicines from the ports to health posts throughout disease-endemic areas in the country [14]. Today, over 146,000 communities and their 4,781,181 CDDs (“foot soldiers”) are committed to completing the final steps in the MDA process after the national health services have supplied medicines to the community health posts. The final steps in the process include taking on some of the costs, especially opportunity costs, such as the time taken away from agricultural activities that they may otherwise be engaged in; collecting IVM from the health posts; and treating all eligible members of their communities [14].

### Who are the CDDs?

The CDDs are members of the disease-affected community. They are selected by whatever manner the community chooses. The selection includes identifying individuals that the community trusts to serve them and who they consider capable of carrying out the necessary activities, including recording the details of MDA [26,27]. CDDs volunteer time and effort, often at a considerable cost to themselves, treating all community residents eligible for MDA [27]. The CDDs who originally committed to distribute IVM were unpaid volunteers, who were trained by personnel of the local Ministry of Health at a central location as determined by the HWs. In a review of the factors that affected the motivation of community members to work as CDDs by Krentel and colleagues [28], training was mentioned by CDDs as the one positive incentive to become a CDD, although they noted that it could also result in financial losses when they left work to attend training sessions. Proper training was also recognised to be essential to enable CDDs to properly conduct their work. Given the distance of the central location from where the CDDs lived, CDDs were often provided with money for transport to the training location or had their travel costs reimbursed. They were also provided with snacks during the training.

The primary occupation of most CDDs delivering NTD drugs has been identified as subsistence farming [28]. The detailed tasks of CDDs have been documented in several publications [27,29,30] and are summarised below.

A CDD

➢ goes for training (with the training venue sometimes quite a distance from his/her place of residence);
➢ receives and signs for the medicines for distribution and presents them to the community leader (and usually takes care of the storage);
➢ liaises with the community leader to provide information on when distribution will start;
➢ provides health education and sensitisation to community members;
➢ registers or updates registration of household members;
➢ goes from house to house distributing the medicines;
➢ repeats house visits targeting absentees and refusals;
➢ records treatment of each eligible person and later records any side effects among the treated people;
➢ gives a summary of treatments using a reporting template (although in some places this is done by the HW);
➢ takes the records of treatment with registration to the HW (although sometimes the HW comes to pick this up); and
➢ reassures and/or refers those suffering from adverse events after treatment.

Chami and colleagues [30] provided an analysis of the characteristics of “best-performing” CDDs and concluded that evidence-based guidelines were required for the selection of CDDs (or community medicine distributors—CMDs [as referred to in that publication]) as well as monitoring their performance. The authors suggested that treatment rates achieved by individual CDDs would be the most useful performance indicator and noted that selection of friends of CDDs could be a useful means of choosing high-performing additions or replacements as friends of CDDs often played a key role in informing other community members about available treatment.

CDDs, as a product of community engagement and participation, influence the community leadership to enquire when medicines for the next cycle of IVM treatment will arrive if they think the time for the next distribution is overdue. CDDs also initiate mobilisation for the collection of additional stocks of medicines when shortages are experienced during distribution [31]. They assist in mobilising the community to adopt bylaws that will ensure treatment compliance [30].

Training sessions are organised and conducted for CDDs prior to MDA at the community or school levels annually. The sessions last for 2 days for new CDDs, and sometimes, a role-play session is included. The training is compulsory for new CDDs and given in a local language/dialect of the CDDs [27]. Depending on the availability or a late arrival of funds, training of CDDs may not be carried out in a given year [29]; the duration may be shortened, or the programme may decide to conduct targeted training—i.e., training/retraining of new and previously trained CDDs who did not perform well during the monitoring of NTD drug distribution. The process of training CDDs enhances the effectiveness and quality of education of CDDs on NTD control methods. All CDDs are trained to

➢ understand and appreciate the meaning of approved and proven correct health education messages they should pass on to community leaders and members;
➢ distinguish between eligible and non-eligible persons for treatment and administration of medicines using a measuring device (dose pole); record all treatments including the number of tablets given to each eligible person; recognise side effects of the different NTD medicines; record and give advice to those with side effects and to refer them when appropriate to health facilities; and
➢ update census information in treatment registers provided by the programme or community [27]. In some NTD programmes, CDDs are trained to record disability observed as part of the integration with morbidity management [29,32].

The treatment registers are the primary sources of information on MDAs; therefore, there is emphasis on quality assurance. The CDDs liaise with the frontline health facility staff to ensure that all records are completed in a timely fashion and kept safely at designated locations.

### Time CDDs put into their work

It is almost impossible for outsiders during short visits to comprehend the complex politics and unique perspectives of rural African communities and their members. Members of remote rural communities do not have an approach to life that requires them to give precise information on distances travelled in units of measurement, such as kilometres, or time taken to undertake their activities and, consequently, they are often not able to provide such exact details [33,34]. Information gathered therefore maybe speculative, although it can be a basis for making reasonable evidentiary assessments. Estimates obtained from an independent mission [34] assessment of CDD work in Liberia and Nigeria on distances walked indicate that in Nigeria, for example, the daily distance walked by CDDs ranged from 2 km by a CDD operating in a semi-urban environment to an average estimate of 10 km/day for CDDs serving a community with widely dispersed households. The median of 13 CDD distances travelled was an estimated 6 km per day during distribution activities, mostly covered on foot. With respect to time, Nigerian CDDs reported working an average of 96 hours per year to complete their tasks, with some spending up to 219 hours. A few individuals had to work up until 10:00 PM, often in complete darkness, because community members were inaccessible during daylight hours [34].

In Liberia, Tanzania, and Cameroon, CDDs reported walking a range of 1 to 4 km per day for 2 to 4 days for distribution of IVM tablets, followed by 5 to 10 km for another few days during follow-up activities. As an example of the travails of some individuals, in an extreme case, one village in Liberia could only be reached after a 5- to 8-hour trek. Half of CDDs in Liberia reported taking about 14 to 21 days in total annually for their distribution work; the other half estimated they took 7 to 10 days [34]. This may explain the high attrition rate witnessed in Liberia as shown in Table 3.

Irrespective of whether the distribution of drugs is annual or semiannual, CDDs, literally foot soldiers in the fight for the elimination of NTDs, take a period of 1 to 4 weeks to complete a single MDA [35,36], depending on the population/CDD ratio, the number of villages/hamlets to be covered, the expanse/topography of the area, and population movements. During project evaluations, community members provide feedback indicating their appreciation of the benefits arising from community volunteers providing medicines for NTD and items for other health issues. For example, as reported in the WHO Progress Report for 2010 [37], a community member from Tanzania stated that

“Before this programme started there was no such thing in the village as doing health work by the villagers. The only community work we had then was to help dig roads. Having used this new method through the APOC, today we can organise ourselves to use this same system to do work in the environment i.e. sanitation; care for our chronically sick people, carry out home-based care for HIVAIDS patients; participate in … sensitizing our community. We have found out that when we go to give ivermectin it is best to do other things related to health at the same time.”–Community Member, Luandai Sone village, IDI, Tanzania [37]

### Output/outcome from CDDs

The average treatment coverage of all 27 countries between 2000 and 2013 was 68.6%. The highest average annual coverage of 83.4% was in Burkina Faso, which had a ratio of 1 CDD to 119 persons in the at-risk population during that period. This was followed by Togo with 83.2% average annual coverage. Togo had a ratio of 536 persons at risk: 1 CDD/HW following the introduction of CDDs to complement the overstretched HWs. DRC with a ratio of 262 persons at risk:1 CDD/HW had an average annual coverage of 61.2% for the same period. It is also important to note that civil unrest and poor infrastructure are factors that significantly hamper treatment coverage in DRC.

**Fig 4:**
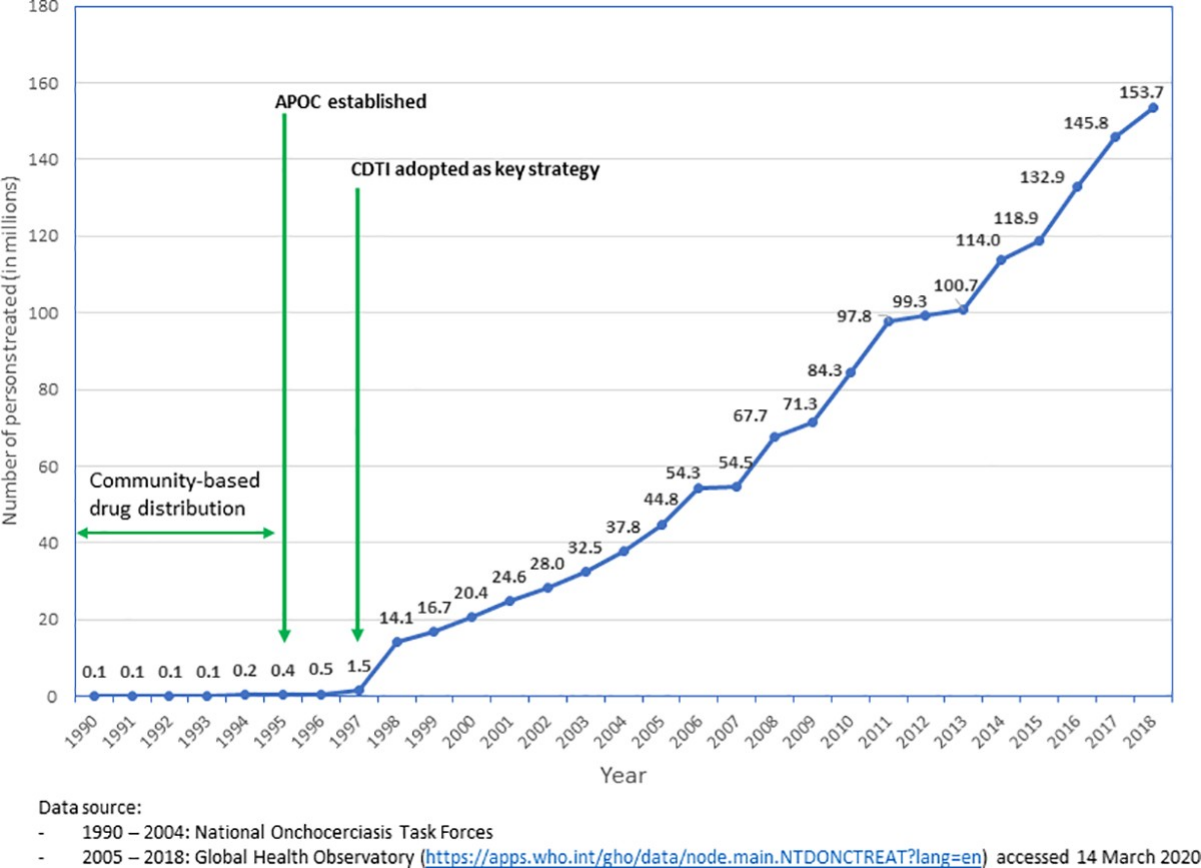
Increase in treatment coverage with involvement of CDDs (1997–2014). APOC, African Programme for Onchocerciasis Control; CDD, community-directed distributor; CDTI, community-directed treatment with ivermectin.

Generally, therefore, as shown in Figs 2 and 3, the higher the ratio of at-risk population to one trained CDD, the lower the average annual coverage with IVM treatment. In Fig 3, the pairwise comparison of means using the least significant difference shows that when the ratio of population per CDD/HW is less than 250, the average therapeutic coverage achieved (72%) is significantly higher (*P* = 0.105) than when this ratio is greater or equal to 250 (62.5%). This suggests that the more CDDs involved in MDA, the lower the workload of the HWs and the greater the number of people who received services, such as mass treatment with IVM. An increased ratio of CDDs to the at-risk population led to a dramatic increase in geographic and treatment coverage rates. This was observed from 1997 when communities and their CDDs took over the distribution of IVM from the health system and, subsequently, the distribution of other NTD medicines (Fig 4).

From the time CDTI started in 1997 to 2010, CDDs distributed almost 2 billion (1,816,138,200) IVM tablets in 16 countries [38] and treated 138,448 communities. Instructively, 40% (55,359) of the communities treated were in post-conflict countries, and the remaining 83,089 communities were in stable countries. The average geographical coverage was 96%.

A CDD from Cameroon expressed a common perception of CDDs towards their work during an assessment of the MDA programme in that country as follows: “…if government refuses to pay we will continue because we are the community. The only problem will be if government fails to bring the drug. Volunteering four days to serve our community is not too much. That is why they selected people from the same community to serve in their communities, so that CDDs will have human feeling for their people” (A CDD speaking on behalf of other CDDs in Fotabong Health Area, Southwest Cameroon [39]].

## Gender and treatment coverage in mass drug administration

There are conflicting reports and publications on treatment coverage differences achieved by male and female CDDs, and some studies have indicated that treatment coverage is better in communities where the proportion of female CDDs is high [40,41].

**Fig 5:**
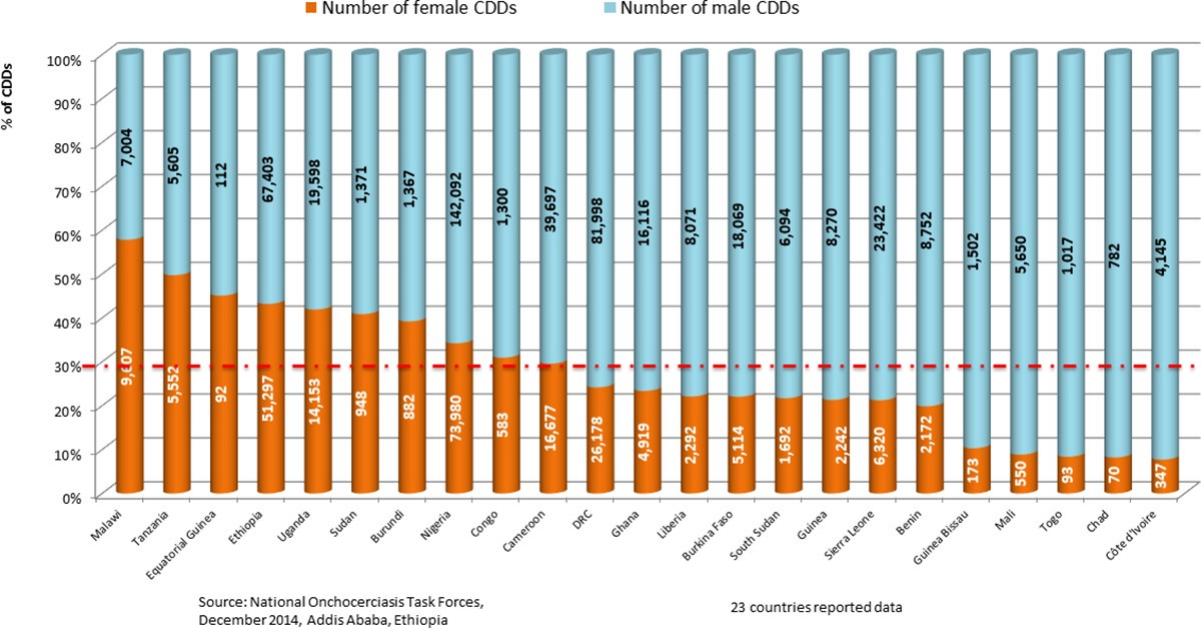
Percentage of female CDDs by country in 2013. CDD, community-directed distributor; DRC, Democratic Republic of Congo.

**Fig 6:**
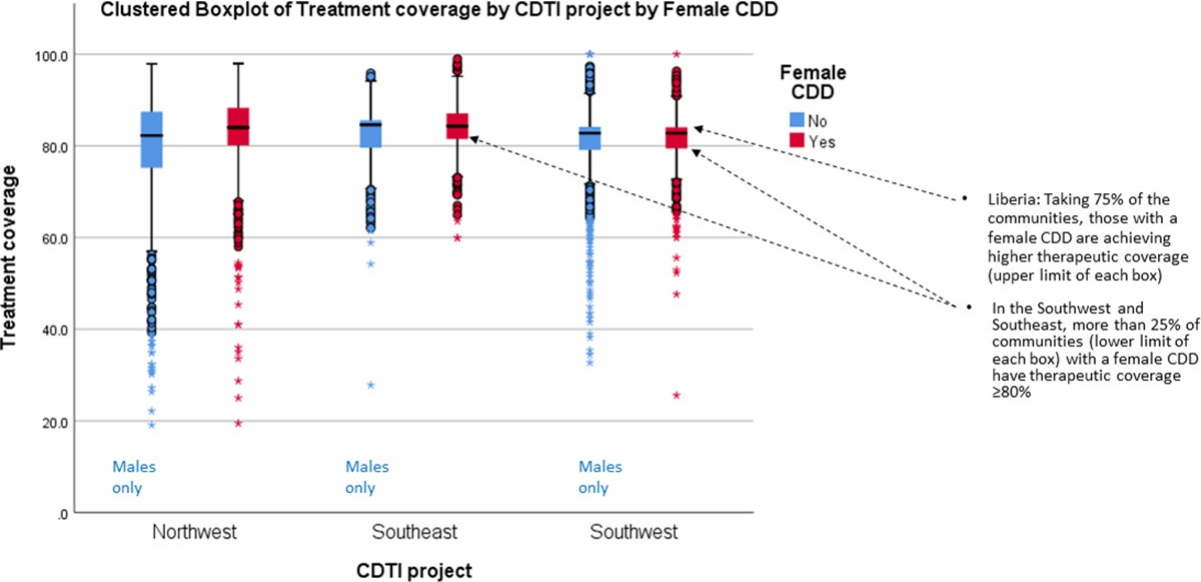
Effect of the participation of female CDDs on therapeutic and geographic coverage in Liberia (2011). CDD, community-directed distributor; CDTI, community-directed treatment with ivermectin.

**Fig 7:**
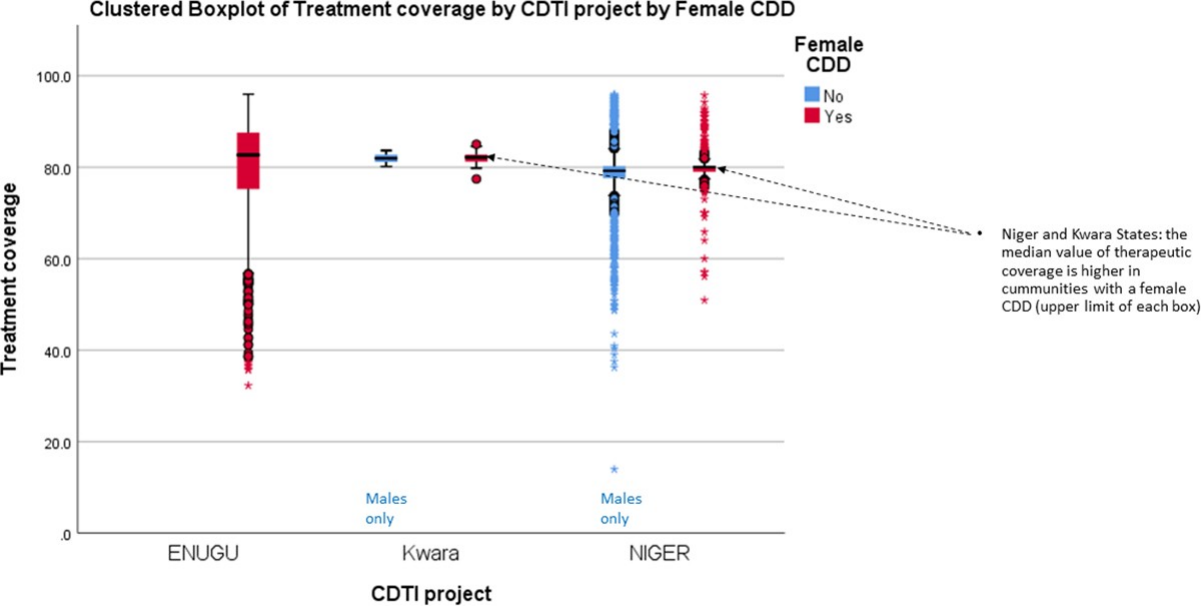
Effect of the participation of female CDDs on therapeutic and geographic coverage in 3 Nigerian states (2011). CDD, community-directed distributor; CDTI, community-directed treatment with ivermectin.

**Fig 8:**
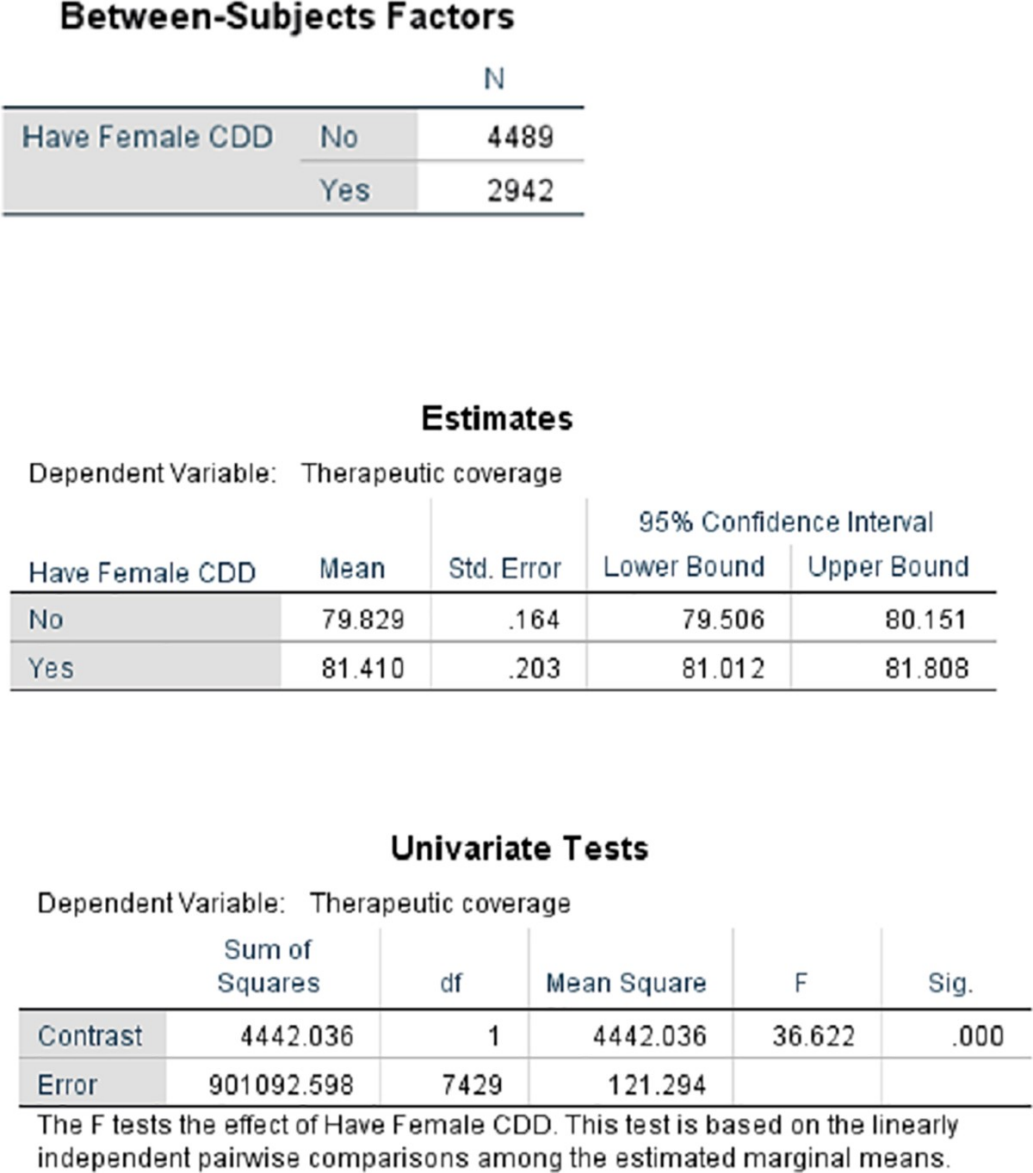
Comparison of therapeutic coverage with and without female CDD in Liberia (2011). CDD, community-directed distributor.

**Fig 9:**
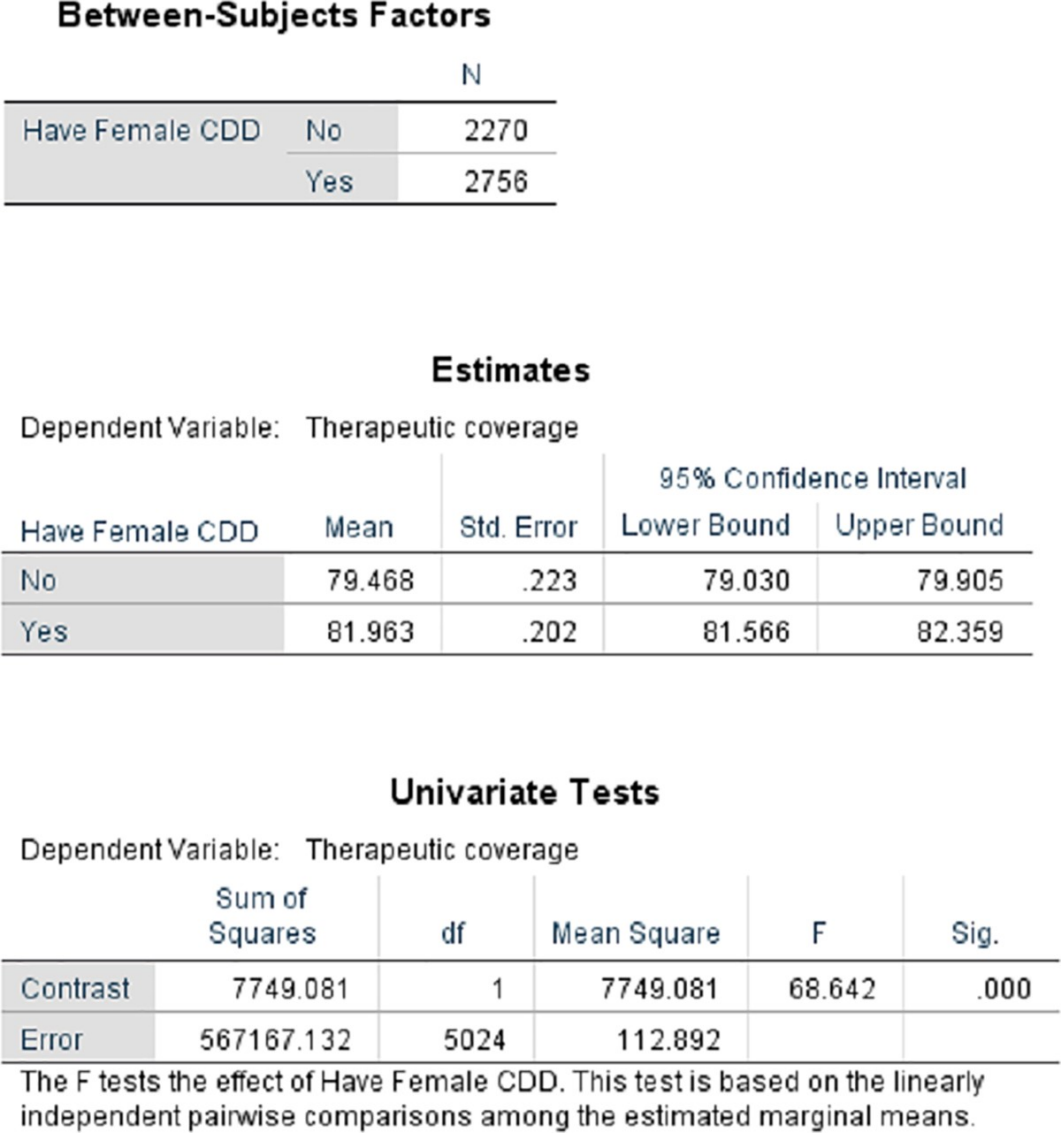
Comparison of therapeutic coverage with and without female CDD in 3 Nigerian states (2011). CDD, community-directed distributor.

Fig 5 presents the proportion of male to female CDDs in 23 countries that provided information in 2013 and the wide variation that occurs regarding these proportions. However, conclusions based on pooled data in Fig 5 may be misleading. The results of disaggregated data by country (rather than pooled regional data) provide evidence from Liberia (Fig 6) and 3 states in Nigeria (Fig 7) underscoring the suggestion of Katabarwa and colleagues [41] that higher treatment coverage may occur with a higher proportion of female CDDs. Thus, Figs 6 and 7 demonstrate a positive correlation between increase in female participation and high treatment coverage. As shown in the boxplot, the top 75% of communities with at least 1 female CDD (upper limit of red coloured boxes) are achieving higher therapeutic coverage than the top 75% of communities that do not have a single female CDD (upper limit of blue coloured boxes). It should be clear, however, that other confounding factors may influence coverage, such as religious beliefs or level of education. Boxplots of Figs 6 and 7 are supported by the analysis of variance presented in Figs 8 and 9 that show that the mean therapeutic coverage is significantly higher (*p* < 0.001) when there is at least 1 female CDD in both Liberia and Nigeria. When there is a female CDD, the mean therapeutic coverage is 81.4% in Liberia and 81.7% in Nigeria versus 79.8% and 79.5%, respectively, when there is no female CDD. Including female CDDs makes it possible to achieve at least the 80% therapeutic coverage, which is required to help attain the elimination of onchocerciasis transmission.

**Fig 10:**
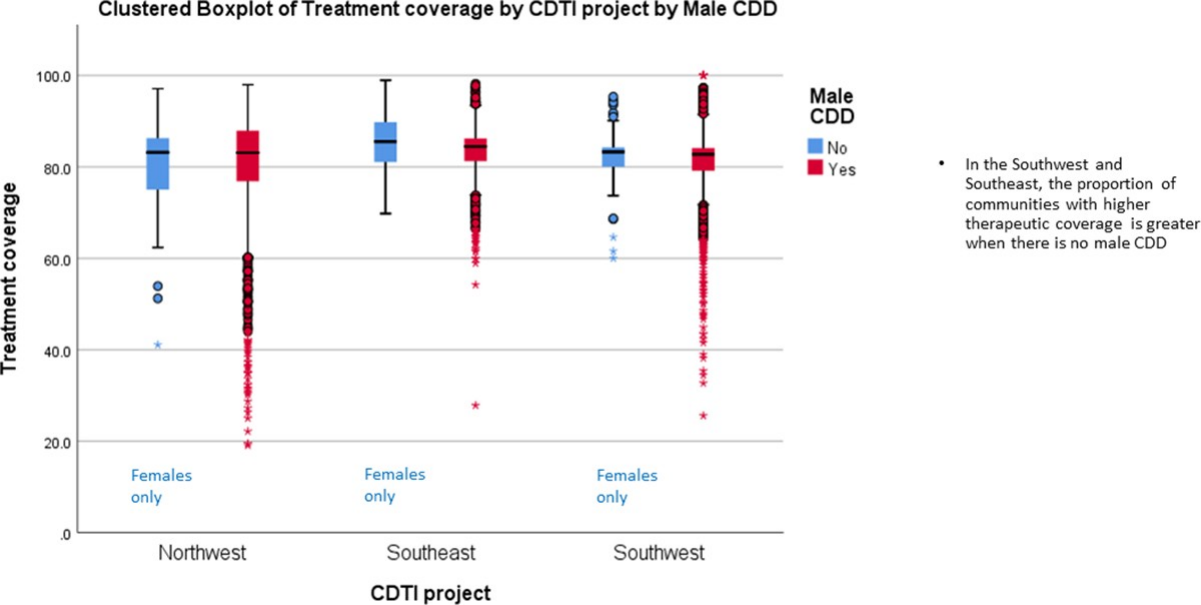
Effect of the participation of male CDDs on therapeutic coverage in Liberia (2011). CDD, community-directed distributor; CDTI, community-directed treatment with ivermectin.

**Fig 11:**
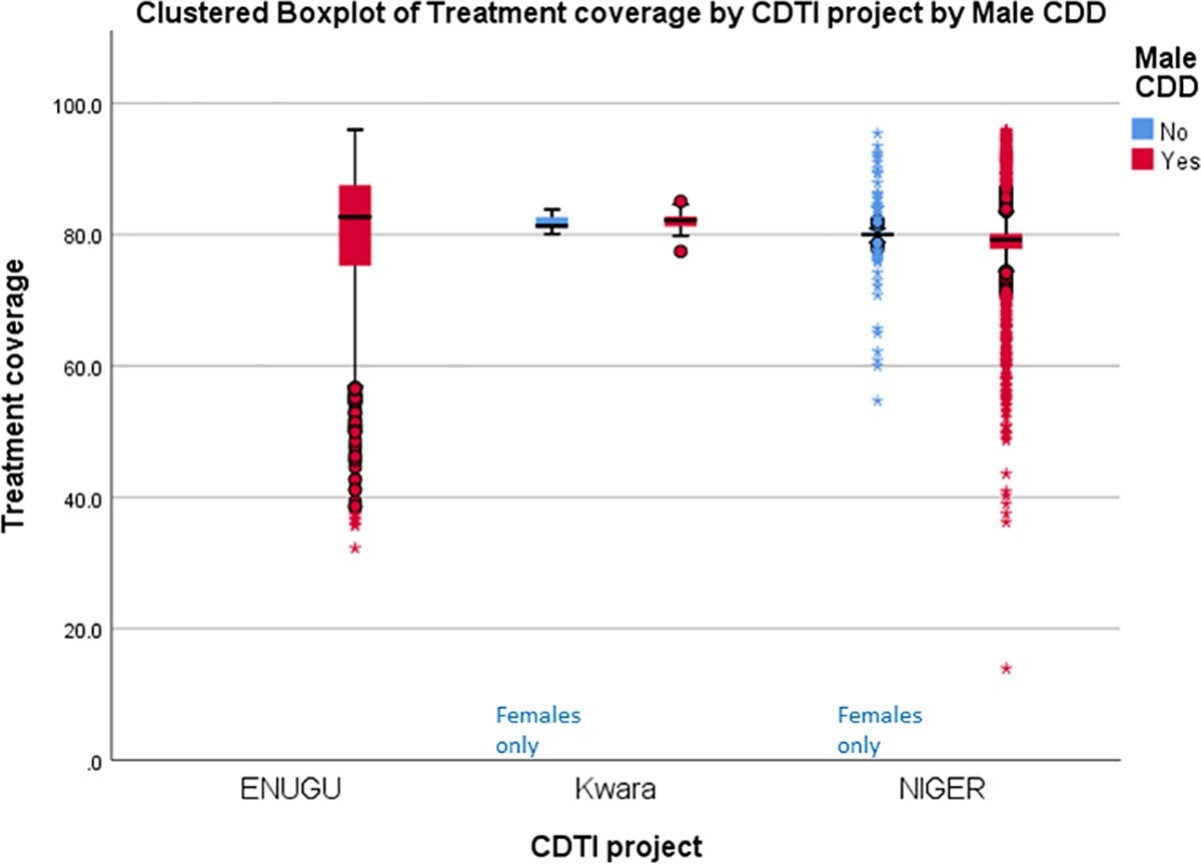
Effect of the participation of male CDDs on therapeutic coverage in 3 Nigerian states (2011). CDD, community-directed distributor; CDTI, community-directed treatment with ivermectin.

Therefore, selecting female CDDs is more likely to contribute towards achieving a higher therapeutic coverage than not having any female among the CDDs in the community. Conversely, the boxplots for the presence of a male CDD (Figs 10 and 11) did not show a significant/positive increase in treatment coverage.

### Community-directed distributors and co-implementation of NTD programmes

**Fig 12:**
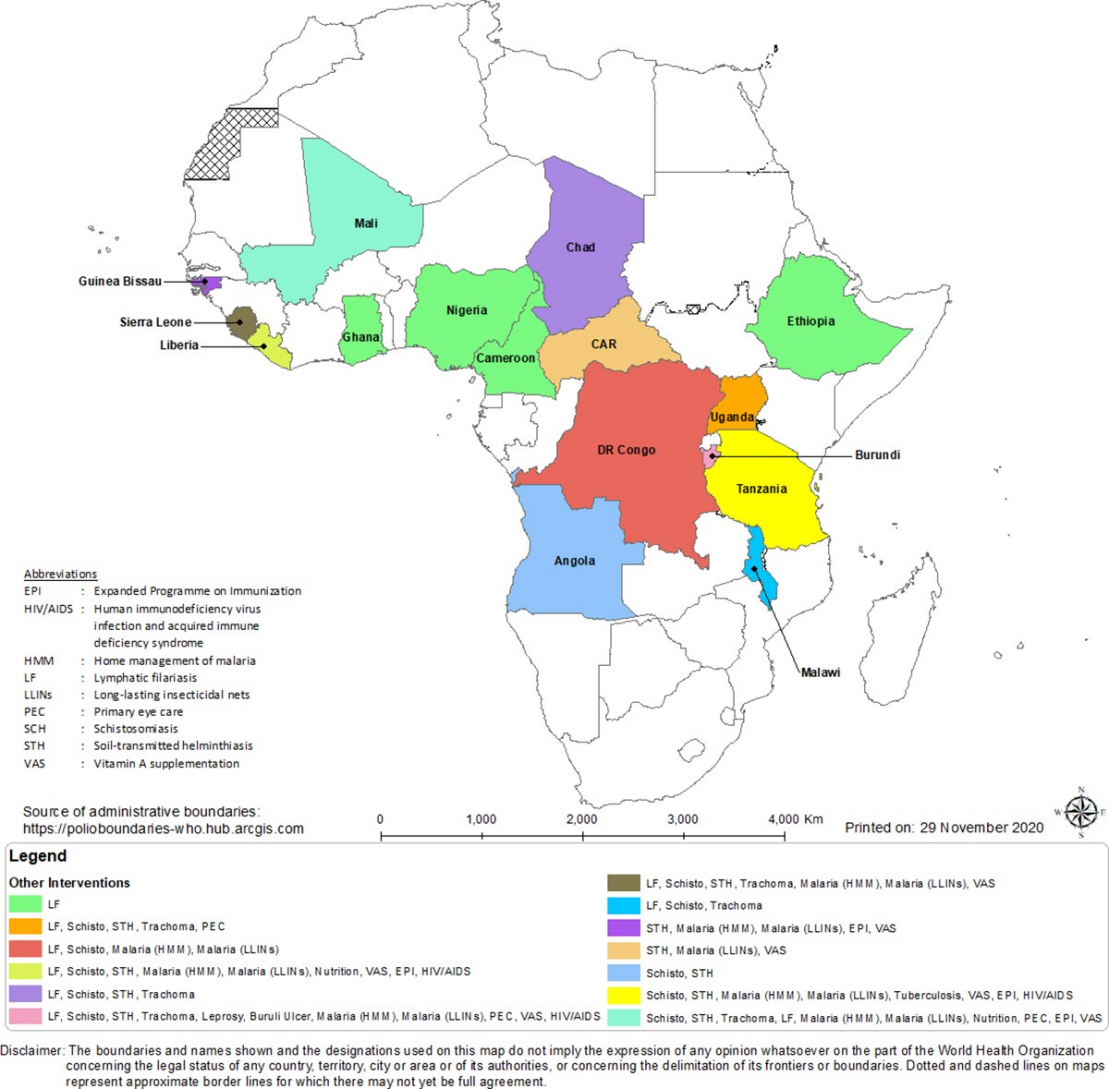
Non-onchocerciasis-related health interventions delivered by CDDs in 16 countries (2000–2012). CAR, Central African Republic; CDD, community-directed distributor.

What is significant about CDDs is not just the huge number of treatments they delivered for onchocerciasis control (194 million treatments in the 23 African countries in 2018 for which data were available) but the other interventions for which a significant proportion of them were engaged (Fig 12). The success of the CDDs in distributing IVM to combat onchocerciasis was quickly recognised and from the year 2000; the CDD system has been exploited to promote and administer numerous other beneficial health interventions [42,43]. Specifically, the fight against NTDs has utilised CDDs to strengthen many interventions by health systems in sub-Saharan Africa. In Nigeria, the Guinea Worm Eradication Programme used CDDs from the CDTI structure for surveillance while preparing the country for certification of successful eradication [44].

**Fig 13:**
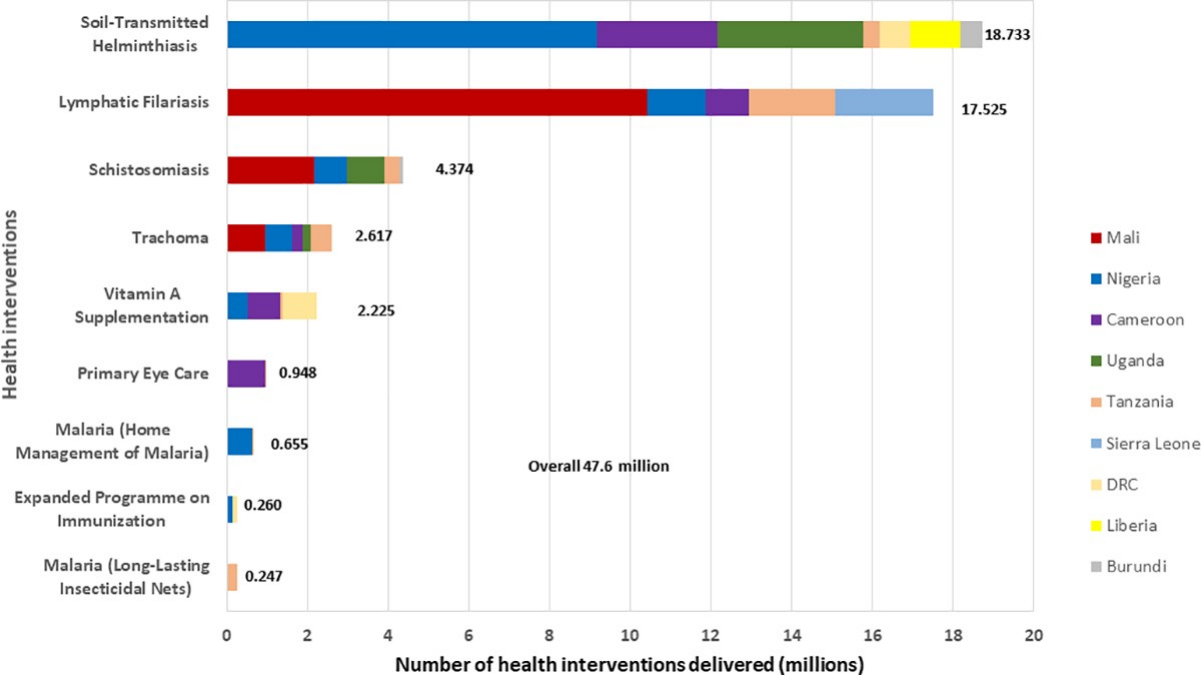
CDDs involvement in the administration of non-onchocerciasis-related health interventions in 9 countries in 2012. CDD, community-directed distributor; DRC, Democratic Republic of Congo.

CDDs have been engaged for such interventions as Home Management of Malaria (HMM); drug treatments for LF, schistosomiasis, and STH; distribution of insecticidal bed nets; and directly observed treatment, short-course (DOTS) drug treatment for TB, as widely reported [45–47]. Similarly, Fleming and colleagues [36] further documented the engagement of CDDs as health mobilisers, for sanitation and hygiene education, HIV/AIDS interventions, TB awareness training, and for polio and immunisation campaigns. By undertaking these additional tasks, CDDs provide valuable extensions of the healthcare delivery system to millions of people living in both accessible and inaccessible areas. Between 2000 and 2013, 4.8 million community members were active as CDDs, and many were involved in delivering multiple health interventions. For example, as shown in Fig 13, in 2012, CDDs reached 47.6 million persons cumulatively with other interventions in 9 countries (Nigeria, Uganda, Tanzania, Sierra Leone, Mali, Liberia, DRC, Cameroon, and Burundi).

This co-implementation has brought about tremendous improvement in programmes such as the LF elimination campaign, efforts to control STH, schistosomiasis, HMM, and immunisation, among others. In Uganda, the use of CDDs proved effective and a feasible option to deliver sulfadoxine-pyrimethamine (SP) for Intermittent Preventive Treatment of malaria in pregnancy (IPTp), because it uses existing community structures and volunteerism [48].

Similarly, the 4.8 million community CDDs in the 27 countries of the WHO Africa Region offer the potential to augment the work of the overstretched health personnel at the community level during this Coronavirus Disease 2019 (COVID-19) crisis. Simply put, because CDDs are trusted and selected by their peers, following adequate specialised training and preparation, they can mobilise and educate community members on the Severe Acute Respiratory Syndrome Coronavirus 2 (SARS-CoV-2) virus, how it is spread, disease symptoms, how to avoid infection, and the process of self-isolation. CDDs could use the NTD or census registers to record the number of sick persons per household and provide other simple but valuable COVID-19-related information.

Co-implementation has also brought about some challenges. The structure of typical rural communities in countries such as Uganda and Nigeria, underpinned by strong kinship ties, has been of historical benefit to the implementation of CDTI, establishing trust and delivering multiple health interventions. However, urbanisation, migration, and an increase of security in conflict areas have led to community structures becoming more diverse and, in some areas, weakening family and kinship ties [49,50]. This has presented challenges for the NTD programmes in establishing, maintaining trust, and scaling up especially in less homogenous settings. Co-implementation of multiple interventions has increased the expectation for incentives from community leaders in exchange for their cooperation. Since sensitisation is key to successful MDA, in communities where the programme has been ongoing for quite a long time, there is implementer fatigue relating to the rigour required for sensitisation and engagement for additional interventions of the various sectors within the community. Other challenges and limitations include misinformation on the purpose and origin of additional NTD medicines, side effects, shifting community priorities, and how to deal with migrant and nomadic groups [50].

The achievements of CDDs that led to the elimination of LF in Togo [6], as well as interruption of onchocerciasis transmission in Nigeria’s Kaduna, Nassarawa, and Plateau states [8], underscore the value of a formal adoption of the CDD mode, and, especially, use of the CDD network in countries with a significant health workforce crisis. It is also important to state that not only has disease elimination been achieved in some foci but also about 500,000 disability-adjusted life years (DALYs) from co-endemic STH infections, LF, and scabies have been averted on account of the work of CDDs [51].

Krentel and colleagues [28,52] and others reviewing control programmes carried out in Haiti, India, Sri Lanka, Tanzania, Sierra Leone, and Indonesia have commended the work of community distributors. While CDDs have been recognised for their contribution to MDA, especially in controlling onchocerciasis and for additional health interventions, there remain many opportunities to engage communities and for wider recognition of their contributions. For example, CDDs are expected to be compensated by the communities that selected them. Such compensation may be in various forms—monetary incentives, exemption from community taxes/labour, and provides recognition at communal functions, etc. However, in some communities, these do not happen. The programme utilises them and reimburses their transport during training and sometimes provides other things such as farming tools to enable them do their work. The governments and partners document the numbers of CDDs trained and utilised and provide other statistics regarding CDDs when reporting on their achievements or publishing papers. On a few occasions out of tens of thousands of CDDs, a few are selected as best performing and given awards or other forms of recognition. At such ceremonies at national and international levels, the focus is usually on the special or outstanding work done by this selected few. CDDs constitute a vital invincible but somewhat not visible force that gets the work done while the credit is frequently shared by the programme, partners, and donors that provided the financial or material support.

WHO has initiated the Roadmap for the elimination of NTDs by 2030. It is critical to harness the unique contribution of CDDs, in this endeavour.

Between 1995 and 2010, MDA with IVM averted 8.2 million DALYs due to onchocerciasis in countries, and it was estimated to have prevented another 9.2 million DALYs between 2011 and 2015 [53]. This remarkable impact of mass treatment by CDDs in remote populations clearly justified the investments in the network of CDDs. Instructively, Fleming and colleagues [36] observed that if this category of stakeholders is not appropriately supported and appreciated, then the Roadmap and any disease control programme are unlikely to succeed.

### Challenges of the CDD model

The success of MDA for NTDs should not suggest that there are no difficulties with the CDD model or that having “foot soldiers” at the community level is the simple solution to all health problems. Providing medicines to a community and attempting to control diseases, particularly vector-borne diseases, is a complex process. There have been unforeseen difficulties with onchocerciasis control that emphasise the need for constant monitoring and surveillance. For example, in the Mahenge endemic focus in Tanzania, after 19 years of annual treatment of the population, there remains a prevalence of the disease almost similar to that found in the 1960s [54]. Lack of consistency of annual treatment and low coverage were reported as important underlying factors. Similarly, in Cameroon, despite more than 15 years of CDD distribution of IVM, a recent study found that there was still a high prevalence of the disease in some areas and concluded that there was still weak community participation and ownership. This weak participation and ownership, together with failings in the timely supply of IVM to the communities, may explain the finding that treatment coverage was lower than reported and why adherence to treatment was not as high as it should have been. The study authors therefore recommended the necessity for reinforcement of community participation and ownership by Ministry of Public Health officials and that speedier procurement of IVM should be facilitated when requested, so that it arrives on time for distribution by the communities and that this would then lead to elimination of transmission [55]. It has also been argued that there is almost no clear delineation of the responsibilities of CDDs nor an updated practical guide for their use. This is essential in the ever-changing NTD landscape in order to guide programme managers, and its absence may have impaired the effectiveness of NTD interventions [56]. The authors suggest that gaps in policy and implementation guidelines on training, supervision, and support to CDDs be addressed in appropriate WHO global and regional documents. It is suggested that delineation of the responsibilities of communities should be clearly set out and that the practical guide for CDDs [27,28,56] should be updated.

Parker and colleagues [57,58] have reported unforeseen difficulties resulting in low treatment coverage for onchocerciasis control and other NTDs and emphasised the need for constant monitoring, evaluation, and surveillance of MDA. In 2012, Allen and Parker [58] referred to several other challenges to control or elimination of NTDs using CDDs including the potential hazard of undermining “fragile and overstretched” health systems with internationally funded vertical programmes. This challenge is less pronounced in settings where the engagement of the CDDs is integrated in the national drug delivery system. Another significant challenge resulting in low coverage is the overreliance by national programmes upon volunteers (CDDs) without sufficient investment in building their capacity.

National programmes rightly place emphasis on integrated platforms for delivery of NTD medicines and tools. However, they allocate insufficient time and resources in continuing training, retraining, and health education for both frontline HWs and for CDDs as well as sensitisation of community members. Given the frequent deployment and turnover of health personnel, NTD programmes need a defined procedure for regular engagement of communities and frontline health staff in decision-making, participation, ownership roles, and responsibilities.

Another important challenge of the CDD model and co-implementation of NTD interventions recently highlighted by Dean and colleagues [59] and others [60,61] is the gender barrier in MDAs. Reasons for the disparity and treatment coverage being significantly higher in areas where women are included as CDDs may relate largely to cultural and religious barriers. However, the principles of gender equity need a better understanding, especially in heterogeneous communities and in the context of the multiple MDA tasks of CDDs.

### CDDs’ perspectives of their challenges in MDA service

It is the presence and availability of CDDs that make treatments of huge populations possible, reaching communities that it had not previously been thought possible to reach by any mechanism of the health service [28,33]. Despite the invaluable role that CDDs have played or are playing, CDDs are often seemingly inadequately appreciated by both community members and national health services.

Under the CDT strategy, communities are expected to provide incentives to the CDDs they have selected. The literature is replete with examples of various forms of incentives, ranging from monetary to in-kind incentives, such as T-shirts, bags, hats, boots, ID cards, bicycles, and waterproof coats that CDDs receive in return for their work [28]. These may be provided by communities or external partners, such as NGDOs and Ministries of Health. Another incentive that has been reported is the giving of preferential treatment to CDDs at district hospitals or health centres [36,61,62]. The types of incentives are to be determined by the communities themselves, and in reality, communities give more in-kind incentives than cash inducements [63].

Several studies have documented a lack of or inadequate community appreciation of the work that the CDDs continue to provide [40,64–66]. According to monitoring reports, community members have defaulted in reimbursing transport or other costs for materials/supplies they were supposed to procure in the first place [64]. In Nri community, in Nigeria, one long-serving CDD argued, “We have worked for 14 years. We are asking for payment (incentives) because other (programmes) are paying us after working for them” [34]. The challenges of monetary incentives and reimbursement to selected programmes in the same community from external and international sources [65] are yet to be resolved. Immunisation programmes have long paid their workers, causing friction and competition within the CDD workforce.

Attrition of CDDs has been linked to the lack of financial incentives or alternative employment opportunities, as well as being due to the long distances trekked during the house-to-house distribution and the time needed for marital and household duties [64,66]. In 2015, da-Costa Vroom and colleagues [64] observed that CDDs earned relatively less income because the MDA period frequently coincided with both the cocoa harvesting and the small-scale gold mining periods, both of which provide handsome financial returns.

In addition to the loss of revenue, distributors incur out-of-pocket expenses in the course of doing the MDA work on account of transportation to training or to collect drug supplies, food and transport during MDA, and purchase of materials for record keeping [36,52,63]. The inaccessibility of many villages means that CDD work can only be done on foot. In Cameroon, a HW observed that “not even bicycles could pass many areas,” and this is clearly the case in many villages affected by onchocerciasis and LF, especially in the rainy season [39]. As an illustration of this problem, some CDDs in Nigeria paid NGN200 to NGN500 (US$0.70 to US$1.38) for motorcycle rides to reach outlying villages. Overall, it has been estimated that the CDDs receive about a 10th of the total value of the “opportunity costs” they incur [36].

In instances where the community determines the period of treatment, dates are often dependent upon the availability of the medicines. CDDs who are farmers may find themselves in difficulties when the treatment period coincides with planting or harvesting times. Several publications have listed the high opportunity costs incurred by CDDs in the process of ensuring that their community members receive donated medicines. The loss of income from these opportunity costs results in household costs for food and school fees of a CDD not being catered for [36], while the CDDs may also forego their own household duties to do MDA work.

Based on interviews with CDDs, health centre staff, and local officials, the top demand of CDDs was for identification and status items, such as branded bags for carrying NTD drugs, uniforms, caps, shirts, or rain capes emblazoned with a symbol of the programmes, such as Mectizan, NTD, or APOC. CDDs prize the status and recognition conferred by such items, but functional concerns are also very important. “When the rain is falling, it will fall on us. And the sun, it is beating on us,” said a CDD in Okpunu Egbu, asking for rain boots, umbrellas, and a hat (male CDD, IDI, Umunachi Community, Nigeria [34].

CDDs have also complained that some HW have considered them to be too materialistic when demanding increased incentives and have consequently failed to provide CDDs with assistance following the occurrence of cases of adverse events [63,67]. Issues have also been raised regarding how literate a distributor must be to be able to competently distribute NTD medicines. This is complicated by perceived links between literacy levels, integrity, and commitment to the task of MDA that have been raised as gradually emerging key issues, especially now that CDDs are being required to distribute an increasing range of health interventions.

CDDs have sometimes complained that they are often blamed when coverage is inadequate, even when the health service has not assisted in replenishing stockouts experienced during MDA [36], or where there has been inadequate supportive supervision from HWs or inadequate information has been provided to CDDs by the HW [47]. Thus, CDDs often become pawns in the “blame game” should anything go wrong with distribution.

Furthermore, CDDs are expected to work and deliver as successfully as they were doing in the early days of distributing solely IVM, despite the increasing complexity and changing circumstances with regard to drug delivery. Such complexities include the introduction of different reporting formats and deadlines and more sceptical and drug-fatigued communities. CDDs also find noncompliance to treatment challenging [67,68]. A review of community-based MDA for LF control/elimination was carried out by Ames and colleagues [69] who observed that some communities lacked knowledge and information about LF and of the MDA campaigns in their communities. This, it was found, could have an impact on how many community members chose to take medication. The study concluded that the length, timing, level of community, and health system involvement, access to care for side effects, inadequate numbers of drug distributors and supervisors, and motivation of drug distributors influenced overall participation in MDA campaigns.

## Conclusions: Lessons from the contributions of communities and their CDDs and some recommendations

We provide the evidence of the continuing sacrifices and contribution to health improvement made by a large army of community members (including CDDs) who often shoulder the largest burden of NTDs with usually inadequate financial resources to strengthen health services at the community level.

A few lessons from this African model are the following:

1. The achievements of over 4.8 million CDDs—mostly unheralded foot soldiers—working in over 146,000 communities in 27 sub-Saharan African countries for priority health interventions underscore the value of continuing investment in engaging African communities to pioneer, or at least, play a key role in the NTD 2030 elimination agenda and in other challenging large-scale health crises such as COVID-19.
2. A good proportion of CDDs have for 18 years, with or without remuneration distributed medicines and other tools helping towards the elimination of NTDs, to reduce the menace of malaria, TB, HIV/AIDS, and increase the reach of immunisation programmes. This long-term commitment shows communities’ value of ownership of their own health needs and that the strategy boosts motivation of CDDs far beyond financial incentives [70].
3. The data show a positive correlation between increase in female participation and high treatment coverage. Therefore, having a female CDD contributes towards achieving a higher therapeutic coverage than not having any female among the CDDs in the community. However, inclusion of women as CDDs in large-scale remotely based programmes needs a more comprehensive understanding which should include a gender analysis to determine how male and female CDDs approach their work especially in heterogeneous communities, in the context of improving MDA coverages with respect to the ever more complex tasks that CDDs are being expected to perform.
4. It is evident from related publications that the work of CDDs drastically reduces costs associated with the control and elimination of onchocerciasis, other NTDs, and off-target infectious diseases [51,53,55], showcasing the CDD model as a cost-effective health service delivery mechanism. It is necessary, nonetheless, to take into account the opportunity costs incurred by CDDs and the demand for provision of incentives.
5. There is motivational remuneration competition within communities [63], which is an ongoing dilemma yet to be resolved by the national health systems, WHO, and development partners.
6. While this paper underscores the value of the CDD model, the model needs to be reviewed constantly, with respect to goals and operation in order to best cope with evolving disease and health system challenges. This will be best served by exploiting the resources of beneficiary communities. We acknowledge the difficult challenges inherent in engaging communities and enabling their complete involvement, including agenda setting, active, and sustained participation, to create an effective and sustainable CDD model. Parker and Allen [57] have identified some of these challenges that are often related to fears and superstitions arising from misunderstandings and coincidental unrelated deaths occurring during MDA interventions. This underlines the need for fully involving communities using good communication techniques to educate and inform communities from the outset before such programmes take place rather than following a “top-down” approach. Furthermore, the drastically understaffed and underresourced national health services desperately need the CDDs, and until recently, some country programmes have lacked well-defined and consistent MDA strategies and guidelines for the elimination of NTDs.

The 2018 Astana Declaration (Alma-Ata 2·0) identifies the inherent “Health for All” principles of PHC as the driving force necessary for achieving the SDGs and UHC [1,71,72]. The second WHO Africa Health Forum in 2019 [73] recommended 4 central (dominant) concepts and enablers of UHC including strong community engagement which the CDD model showcases.

A review of the contribution of efforts to control NTDs to the SDGs concluded “that progress on alleviating the burden of NTDs is a prerequisite for progress towards SDG 1” [2]. It is unlikely that many African countries will achieve all the SDG goals by 2030. However, the low-hanging fruits—elimination of 5 NTDs—can be achieved by 2030 if national and international health system actors harness the potential of community engagement [74] and all available networks of CDDs.

As Krentel and colleagues [28] noted in their review of factors motivating CDDs, community distributors have, over the years, brought lifesaving and health improving care into the homes of billions of people. Thus, in the African context, CDDs represent a readily available, credible, and tried and tested resource, already embedded in the continent’s remotest communities. The CDDs undoubtedly deserve global recognition and more credit and motivation than they currently receive, after having demonstrated high-quality MDA and other services uninterrupted for over 20 years.

The COVID-19 situation has emphasised the unpredictability of public health in the aftermath of the pandemic. Funds for national health systems in Africa, from both national government and overseas aid, may be increasingly limited for the next few years at least. In such circumstances, where paid employment will be scarce, a large cadre of trained community-based volunteers that already exists may be useful with respect to continuing to deliver essential public health improvements.

We **recommend** the African community-designed implementation of integrated delivery of public goods to improve community health, a system already encompassing over 4.8 million CDDs be reviewed and proactively integrated into the WHO NTD Roadmap for 2030 and adapted by countries and development partners to strengthen national health services. The approach will strengthen and sustain community engagement in NTD interventions especially during the search for new financing mechanisms for NTDs. A WHO policy instrument providing guidance to countries on the adaptation, refinement, and customisation of the community-directed approach [CDD model] for the elimination of NTDs will be important.

Currently, it is forecast that there will be 1.5 billion people requiring interventions against NTDs in 2020, with a target to reduce this to 200 million by 2030. If these targets are to be met, it is critical that everyone, from the topmost levels of the health service to the community level volunteers, work together cohesively, as partners, to realise a common goal. If that can be achieved, there is a good chance that the SDGs, at least those focussing on the NTDs, can be accomplished by 2030.

## Method and source of data for the review

The major sources of data for this review consist of the WHO APOC data from 1997 to 2013, supplemented by progress report of WHO/APOC, reports of countries participating in the OCP, and 74 peer-reviewed scientific articles related to the elimination of lymphatic filariasis and onchocerciasis.

Other sources of information including community and CDD voices and perceptions about their work and challenges were gathered from CDT strategy implementation guidelines, CDD training manual, publications of WHO/Special Programme for Research and Training in Tropical Diseases (TDR) Taskforce for onchocerciasis and lymphatic filariasis, as well as reports of independent participatory monitoring and sustainability evaluations.

We had access to reports of countries to APOC, the national NTD master plan of Nigeria, and a report of WHO Liberia during the Ebola crisis. Four coauthors were staff of OCP and APOC from 1996 to 2015.

## Supporting information

S1 Data Harmonisation of treatment and training by project (1999–2013). (XLS)

S1 Table Supporting information on CDDs who have served in MDA uninterrupted for 10 and 19 years in Ghana, Nigeria (Kaduna State), and Cameroon (southwest region) between 2000 and 2018. CDD, community-directed distributor; MDA, mass drug administration.
(DOCX)
