# Supplementary material for: Community-directed distributors—The “foot soldiers” in the fight to control and eliminate neglected tropical diseases
Source: PLoS Negl Trop Dis. 2021 Mar 4;15(3):e0009088. doi: 10.1371/journal.pntd.0009088 (PMC7932156; doi:10.1371/journal.pntd.0009088)
Supplement: S1 Table — CDD, community-directed distributor; MDA, mass drug administration. (DOCX) [file pntd.0009088.s002.docx]

**S1 Table:** Supporting Information on CDDs who have served in MDA uninterrupted for 10 and 19 years in Ghana; Nigeria (Kaduna State) and Cameroon (Southwest region) between 2000 and 2018.

**Table A in S1 Table:** CDDs who have served for 10 or 19 years in Ghana and Kaduna State in Nigeria between 2000 and 2018

|  |  | **CDDs who have served from 2009 to 2018** | | | | **CDDs who have served from 2000 to 2018** | | |
| --- | --- | --- | --- | --- | --- | --- | --- | --- |
| **Country, region** | **Area** | **Male** | **Female** | **Total** |  | **Male** | **Female** | **Total** |
| **Ghana (regions)** | Ashanti | 1,131 | 416 | 1,547 |  | 221 | 13 | 234 |
|  | Brong Ahafo | 1,143 | 165 | 1,308 |  | 124 | 54 | 178 |
|  | Central | 219 | 90 | 309 |  | 185 | 67 | 252 |
|  | Eastern | 347 | 101 | 448 |  | 119 | 17 | 136 |
|  | Northern | 1,056 | 228 | 1,284 |  | 794 | 42 | 836 |
|  | Upper East | 194 | 127 | 321 |  | 87 | 23 | 110 |
|  | Upper West | 1,013 | 488 | 1,501 |  | 632 | 48 | 680 |
|  | Volta | 541 | 150 | 691 |  | 135 | 14 | 149 |
|  | Western | 1,307 | 415 | 1,722 |  | 200 | 52 | 252 |
|  | ***Sub-total*** | ***6,951*** | ***2,180*** | ***9,131*** |  | ***2,497*** | ***330*** | ***2,827*** |
| **Nigeria, Kaduna State (LGAs)** | Birnin-Gwari | 81 | 14 | 95 |  | 71 | 11 | 82 |
|  | Chikun | 61 | 10 | 71 |  | 53 | 8 | 61 |
|  | Giwa | 15 | 0 | 15 |  | 10 | 0 | 10 |
|  | Igabi | 25 | 0 | 25 |  | 19 | 0 | 19 |
|  | Ikara | 15 | 0 | 15 |  | 15 | 0 | 15 |
|  | Jaba | 46 | 15 | 61 |  | 35 | 9 | 44 |
|  | Jema'a | 55 | 15 | 70 |  | 26 | 12 | 38 |
|  | Kachia | 62 | 11 | 73 |  | 42 | 9 | 51 |
|  | Kaduna North | 0 | 0 | 0 |  | 0 | 0 | 0 |
|  | Kaduna South | 0 | 0 | 0 |  | 0 | 0 | 0 |
|  | Kagarko | 102 | 2 | 104 |  | 64 | 2 | 66 |
|  | Kajuru | 65 | 12 | 77 |  | 48 | 8 | 56 |
|  | Kaura | 157 | 15 | 172 |  | 32 | 3 | 35 |
|  | Kauru | 214 | 5 | 219 |  | 67 | 2 | 69 |
|  | Kubau | 121 | 0 | 121 |  | 23 | 0 | 23 |
|  | Kudan | 0 | 0 | 0 |  | 0 | 0 | 0 |
|  | Lere | 215 | 15 | 230 |  | 57 | 6 | 63 |
|  | Makarfi | 0 | 0 | 0 |  | 0 | 0 | 0 |
|  | Sabo-Gari | 0 | 0 | 0 |  | 0 | 0 | 0 |
|  | Sanga | 56 | 12 | 68 |  | 26 | 6 | 32 |
|  | Soba | 0 | 0 | 0 |  | 0 | 0 | 0 |
|  | Zangon-Kataf | 79 | 26 | 105 |  | 49 | 14 | 63 |
|  | Zaria | 0 | 0 | 0 |  | 0 | 0 | 0 |
|  | ***Sub-total*** | ***1,369*** | ***152*** | ***1,521*** |  | ***637*** | ***90*** | ***727*** |

**Table B in S1 Table:** CDDs who have served for 19 years in southwest region, Cameroon between 2000 and 2018

|  |  | **CDDs who have served from 2000 to 2018** | | |
| --- | --- | --- | --- | --- |
| **Country, region** | **Area** | **Male** | **Female** | **Total** |
| **Cameroon, Southwest region (districts)** | Akwaya | 26 | 13 | 39 |
|  | Bakassi | 17 | 9 | 26 |
|  | Bangem | 27 | 12 | 39 |
|  | Buea | 19 | 28 | 47 |
|  | Ekondo Titi | 31 | 11 | 42 |
|  | Eyumojock | 23 | 15 | 38 |
|  | Fontem | 28 | 15 | 43 |
|  | Konye | 21 | 14 | 35 |
|  | Kumba | 39 | 63 | 102 |
|  | Limbe | 19 | 34 | 53 |
|  | Mamfe | 21 | 10 | 31 |
|  | Mbonge | 22 | 41 | 63 |
|  | Mundemba | 15 | 10 | 25 |
|  | Muyuka | 11 | 23 | 34 |
|  | Nguti | 22 | 13 | 35 |
|  | Tiko | 18 | 41 | 59 |
|  | Tombel | 22 | 11 | 33 |
|  | Wabane | 25 | 15 | 40 |
|  | ***Sub-total*** | ***406*** | ***378*** | ***784*** |
| **Grand Total(2000 – 2018)**  **Ghana, Kaduna (Nigeria), Southwest region (Cameroon)** |  | **3,540** | **798** | **4,338** |
| **Grand Total(2009 – 2018)**  **(Ghana and Kaduna (Nigeria)** |  | **8,320** | **2,332** | **10,652** |

LEGEND

Community-directed distributor

Local Government Authority
